# Comparison of transmission parameters between *Anopheles argyritarsis* and *Anopheles pseudopunctipennis* in two ecologically different localities of Bolivia

**DOI:** 10.1186/1475-2875-12-282

**Published:** 2013-08-13

**Authors:** Frédéric Lardeux, Claudia Aliaga, Rosenka Tejerina, Libia Torrez

**Affiliations:** 1 Institut de Recherche pour le Développement (IRD), CP 9214, La Paz, Bolivia; 2 UMR MIVEGEC, Maladies Infectieuses, Vecteurs, Ecologie, Génétique, Evolution et Contrôle, Université de Montpellier 1, CNRS 5290, IRD 224, BP 64501, 34394, Montpellier Cedex 5, France; 3 Instituto Nacional de Laboratorios de Salud (INLASA), Laboratorio de Entomología Médica, Rafael Zubieta 1889, Miraflores, Casilla M-10019, La Paz, Bolivia; 4 Instituto de Biología Molecular y Biotecnología, Facultad de Ciencias Puras, Universidad Mayor de San Andrés, C 27 Campus Universitario Cota-Cota, La Paz, Bolivia

**Keywords:** Anopheles argyritarsis, Anopheles pseudopunctipennis, Transmission, Bolivia, Vectorial capacity, Entomological inoculation rate, Plasmodium

## Abstract

**Background:**

*Anopheles (Anopheles) pseudopunctipennis* is a recognized malaria vector in the slopes of the Andes of Bolivia. There, other species might be involved in malaria transmission and one candidate could be *Anopheles argyritarsis*. Although it is generally admitted that this species is not a malaria vector in the neotropical region, its potential role in transmission is still controversial and this situation has to be cleared, at least for Bolivia. Comparing the vectorial efficiency of *An. pseudopunctipennis* with that of *An. argyritarsis* could solve the question.

**Methods:**

The two species were sampled throughout Bolivia to estimate their degree of co-existence in their distribution range. Vectorial efficiencies of the two species were compared in two ecologically different localities where the species were sympatric by analysing their vectorial capacities and components (i e, human biting rates, human biting index, survival, durations of the gonotrophic cycle and extrinsic cycle), and the entomological inoculation rates (EIR). Mosquitoes were sampled monthly during more than one year in the two localities. A monthly sample consisted in hourly captures in four houses (inside and outside) in each locality, during four consecutive nights. Climatic variables (temperature, humidity, potential evapo-transpiration and precipitations) were recorded to better understand variability in the entomological parameters. Relationships were analysed using multivariate methods.

**Results:**

*Anopheles pseudopunctipennis* and *An. argyritarsis* are “altitude” species, sharing the same geographical distribution range in the Andes of Bolivia. No *Plasmodium* parasite was identified in *An. argyritarsis* and estimates of the vectorial capacity indicated that it is not a malaria vector in the two studied localities, unlike *An. pseudopunctipennis* which showed positive EIRs. This latter species, although not a very good malaria vector, exhibited better life traits values and better behavioural characteristics in favour of transmission as compared to *An. argyritarsis*.

**Conclusions:**

In the Andes of Bolivia, above 1000 m of altitude, *An. pseudopunctipennis* is likely to be the only malaria vector. There, it is present almost everywhere and priority control effort should be directed toward this species. Below 1000 m of altitude, vector incrimination should also be focused on other sympatric species (likely not *An. argyritarsis*) that might be locally important. From the present study, candidates would be among *Anopheles rangeli*, *Anopheles triannulatus s.l.*, *Anopheles trinkae*, *Anopheles nuneztovari s.l.*, *Anopheles oswaldoi s.l.* and *Anopheles benarrochi s.l.*

## Background

*Anopheles (Anopheles) pseudopunctipennis* has long been recognized a major malaria vector in the foothills and mountainous regions of the Andean countries of South America [1], including Bolivia [2] and its neighbouring countries such as northern Argentina [3]. *Anopheles (Nyssorhynchus) argyritarsis* is less well known. It is a neotropical mosquito which geographical distribution is almost that of *An. pseudopunctipennis*: it occurs from Mexico (state of Guerrero) to the northern part of Argentina in South America [4]. In this wide geographical distribution range, it only seems to be absent from two western countries, Ecuador and Chile, while *An. pseudopunctipennis* is present.

A large amount of controversial literature exists on the potential role of *An. argyritarsis* in the transmission of *Plasmodium* parasites [5]. Early reports on its vector status are confusing and discrepancies are observed amongst published results [6-19]. Facing such discrepancies, the accepted conclusion was that the species could be a potential *Plasmodium* carrier and could be able to transmit the parasites. However, in the 1980s the discrepancy in opinions before the 1930s was found likely to be the result of misidentifications of the species. At that time, it was sometimes probably confused with actual confirmed vectors such as *Anopheles darlingi*, *Anopheles albimanus* or *Anopheles braziliensis*[4,20]. Nowadays, there exists a general agreement on the non-vector status of *An. argyritarsis* in the neotropical region [5], despite a few remaining local issues to be addressed [21]. Unfortunately, even in recent studies, no values for significant transmission parameters, such as the vectorial capacity or the entomological inoculation rate (EIR), were given to sustain such a conclusion. Therefore, the status of *An. argyritarsis* as a malaria vector still needs to be clarified, at least in some regions [22]. This is the case for Bolivia.

In the present study, *An. argyritarsis* will be compared to *An. pseudopunctipennis*. In Bolivia, the two species have an almost similar geographical distribution range, in which malaria epidemics occur. In the country, 10,000 to 30,000 cases of malaria are reported each year, the majority of which (80%) comes from the Amazonian region. However, the foothills and mountainous regions of the Andes, where *An. pseudopunctipennis* and *An. argyritarsis* develop, are still malaria-endemic regions [23], especially in the centre and south of the country where the present study took place.

To compare the relative transmission efficiency of the two species, parameters of their respective vectorial capacity and EIR (which is the number of infectious bites received per day by a human, or equivalently, the human biting rate multiplied by the sporozoite rate [24]) have been estimated monthly in two ecologically different localities where the two species were sympatric. Additional characteristics have been analysed, such as hourly biting patterns and endo/exophagy behaviour which may affect the man/mosquito contact and therefore may help to better understand the differences observed in the vector status of the two species.

## Methods

The study operates two types of data. The first type came from the INLASA mosquito collection database in which, apart from the stored specimens, ecological data on collecting sites are available. These data characterize larval habitats of both species, map their presence in Bolivia and estimate their degree of co-existence. The first following paragraph describes the methods used to analyse these data.

The second type of data consisted of two longitudinal studies carried out in two different localities, Mataral and Caiza, where the two species were sympatric. They consisted of samples of human biting mosquito females and a collection of climatic variables. From these captures, bionomics data have been estimated, and entomological parameters for malaria transmission (components of the vectorial capacity and EIR) have been computed. The second and all the subsequent paragraphs detail these two longitudinal studies.

### Co-existence of the two species in their distribution range

*Anopheles* larvae were collected from 535 larval breeding sites throughout Bolivia. Each site was georeferenced and characterized by means of simple ecological data following [25]. *Anopheles* larvae were mounted and identified using [26]. For the present study, only sites where *An. pseudopunctipennis* and/or *An. argyritarsis* were captured have been retained. To measure the degree of co-existence between the two species over the different collecting sites, the Jaccard index [27] was used. The statistical significance of the Jaccard index was assessed using published statistical tables [28].

### Study areas for the longitudinal studies

The specific longitudinal studies were carried out in two different localities. The first, Mataral (latitude -18.60°, longitude -65.14°, altitude 1500 m) is situated in the centre of Bolivia, in the dry inter-Andean valleys, approximately 100 km north of the constitutional capital Sucre. The second, Caiza (latitude −21.79°, longitude −63.55°, altitude 570 m), is situated in the south of Bolivia, in the Chaco region, approximately 10 km of the city of Yacuiba, border with Argentina. Both localities are small villages of ≈ 100-150 houses. Houses are mostly made of mud bricks (adobe), with thatch or tin roofs. In Caiza, living standards are a little higher than in Mataral: more brick houses are present. People are mostly subsistence farmers. Domestic animals, including goats, sheep, pigs, dogs, chickens, some cows and donkeys wander in the villages. Both villages lie close to a river where *An. pseudopunctipennis* larval habitats are found in the low margins of the rivers [29-32].

In Mataral, the climate is xeric, characterized by a mean annual temperature of 18°C, with mean maximum of 27°C and mean minimum of 9°C. In summer (December-March), this mean temperature may be >35°C and in winter (June-August), the mean minimum may be <5°C. Rainfalls are short and violent and occur mainly between November and March. Their annual mean is between 400 and 600 mm, but in the winter months may be totally dry. The overall mean relative humidity is ≈ 50%.

In Caiza, the climate is semi-tropical/semi-arid, characterized by a mean annual temperature of 21°C, with mean maximum of 27°C and mean minimum of 15°C. Mean precipitations are 1188 mm/year and the overall mean relative humidity is ≈ 70%. As in Mataral, rainfall is more abundant during the hottest months in December-March while winter months are the driest, sometimes totally dry.

In both regions, the National Control Programme for Chagas disease was involved, carrying out indoor and outdoor insecticide sprayings. However, its actions were sporadic and at the time of the study, no insecticide campaign was undertaken in the two villages, or even two years before, leaving mosquito densities evolve naturally.

### Mosquito collections and biting habits

Mosquitoes were captured in both localities using the landing catch technique [33] from March 2005 to June 2006. In each locality, four houses were chosen and each month, during four consecutive nights, mosquitoes were sampled inside and outside each house. Captures were carried out from 18.00 to 06.00 and catches were hourly recorded. For one sampling session (i e, captures carried out each month, during the four nights, in the four houses, inside and outside the houses), endophagy was computed as the overall proportion of mosquitoes biting inside houses. Hourly biting patterns were analysed using the hourly total number of mosquitoes captured during a sampling session. Because there is no marked difference in day/night periods among seasons [34], differences in hourly activities between the two *Anopheles* species were observed by comparing the median hour of activity among the species computed each month.

In the field, mosquitoes were identified using standard morphological keys [26] and were dissected to categorize them as parous or nulliparous from the aspect of their ovaries, parous females being characterized by the absence of skeins in their ovary tracheal system [35].

### Vectorial capacity and entomological inoculation rates

The vectorial capacity describes the transmission potential of a mosquito population in the absence of *Plasmodium*[36] and the EIR is a measure of the intensity of transmission. Therefore, these two indices were used to compare the efficiency of *An. argyritarsis* and *An. pseudopunctipennis* as malaria vectors, using the formulas given in [24]. Other informative parameters can be derived from the vectorial capacity such as: (i) the proportion of mosquitoes that survived the duration of the sporogonic cycle and are therefore “epidemiologically dangerous females” as they might carry sporozoites, or (ii) the expected infective life time [37], which have been estimated in this study.

The computation of these indices needs the estimation of their constituting parameters which are: the human biting rate (HBR), the human feeding rate (*a*), the probability that an individual mosquito survives one day (*p*), the proportion of infective mosquitoes (*s*) and the length of the incubation period of the parasite in mosquitoes (i e., the extrinsic or sporogonic cycle) (*n*) which has been computed using the classical formula of Detinova [35] with constant values of 105°C days for the total number of degree-days to complete sporogony and 14.5°C for the temperature threshold below which the parasite cannot develop. These constant values correspond *Plasmodium vivax,* which is the only species encountered in the distribution range of *An. pseudopunctipennis* and *An. argyritarsis* in Bolivia [38].

### Estimation of human biting rates and relationships with climatic variables

The HBRs were computed for each sampling period using the total number of mosquitoes captured during the sampling session divided by the total number of nights of captures and the total number of sites sampled during the period (a capture site is a “house intradomiciliar” or “house peridomiciliar”). Statistical analysis of relationships between HBR and climatic variables were carried out in each locality using multiple correspondence analysis (MCA) to detect and represent underlying structures in the data set, in particular when non-linear relationships exist amongst the data [39]. The raw data table consisted of 15 (Caiza) or 16 (Mataral) monthly sampling sessions x (climatic variables + HBR results). Variables (climatic and HBR) were split into disjunctive classes (into low, medium and high values) and the disjunctive table was transformed into a Burt table prior to MCA processing. Significant variables were identified by means of their elevated contribution to the inertia of the axes [40].

Scatter plots of HBR values x climatic variables were drawn to detect likely linear relationships whose significance (and slope sign) was tested using the standardized coefficients β for linear regression and its associated *t*-test. MCA and regression analysis were carried out using the Statistica software [41].

### Estimation of survival rates (*p*)

The daily survival probability *p* was estimated using the Davidson’s formula [42]: *p=Q*^*1/g*^, where *Q* is the proportion of parous females in the population and *g* is the duration of the gonotrophic cycle (in days).

For *An. pseudopunctipennis*, the duration of the gonotrophic cycle has been modelled earlier using functions depending on ambient temperatures [43]. This model was used in this study to compute monthly values of *g* in the two studied localities for this species (Table 1). Because *g* is temperature dependant, *p* will also fluctuate according to temperature. In a first approach, the same monthly computed values for *g* were selected for the two *Anopheles* species, and only monthly parous rates computed with a sufficient number of captured mosquitoes (i e, captures >100 individuals) were taken into account to ensure statistical validity of *Q*. The Davidson’s formula assumes that the mosquito population age structure should be stable over the period of data collection. Then, only months when no adverse climatic conditions were observed some weeks before mosquito sampling were taken into account to ensure that the mosquito populations were stable.

**Table 1:** **Transmission parameters for *Anopheles pseudopunctipennis *in Mataral during the study period (March 2005–June 2006)**

**Sampling period**	**Mosq capt**	**HBR**	**Q**	***g***	***p***	**Endo**	**Median hour**	**Mosq PCR**	***s***	***s *95% confidence interval**	***a***	**CV**	**EIR**	***Infec life time***	***Density infective females***
March	691	38.39	0.43	4.2	0.818	0.44	22-23	231	0.45	0.01–2.29	0.071	0.69	17.28	0.3	1.96
April	1635	68.13	0.73	3.9	0.922	0.48	21-22	1572	0.06	0.01–0.33	0.077	22.25	4.09	4.2	23.32
May	1011	25.28	0.51	4.6	0.864	0.39	21-22	978	0.84	0.34–1.69	0.065	1.01	21.24	0.6	2.27
June	1679	52.47	0.53	5.3	0.887	0.39	20-21	2354	0.47	0.22–0.87	0.057	2.12	24.66	0.7	4.49
July	730	17.38	0.40	9.2	0.905	0.52	19-20	727	0.42	0.08–1.21	0.033	0.14	7.30	0.2	0.43
August	573	17.91	0.45	6.4	0.883	0.45	21-22	659	0.47	0.09–1.35	0.047	0.31	8.42	0.4	0.82
September	103	17.17	0.77	3.1	0.919	0.59	23-24	-	-	-	0.096	8.31	-	5.1	7.32
October	1574	49.19	0.40	3.5	0.770	0.40	23-24	1095	0.57	0.19–1.15	0.085	0.81	28.04	0.2	2.50
November	65	2.03	0.77	3.9	0.935	0.51	21-22	62	1.63	0.05–8.16	0.078	0.95	3.31	5.9	0.82
December	59	1.74	0.52	3.6	0.834	0.58	23-24	97	0	-	0.084	0.10	0.00	0.6	0.21
January	1692	56.40	0.72	4.0	0.921	0.36	22-23	1783	0.29	0.09–0.68	0.076	16.99	16.36	3.9	18.42
February	61	2.54	0.62	4.4	0.897	0.46	21-22	58	0	-	0.069	0.28	0.00	1.6	0.44
March	91	3.25	0.90	4.5	0.977	0.33	21-22	114	0	-	0.067	6.49	0.00	29.7	2.27
April	333	9.25	0.87	5.2	0.974	0.29	21-22	338	0.26	0.23–0.35	0.058	12.68	2.41	23.0	5.77
May	1363	37.86	0.59	6.0	0.916	0.41	22-23	2597	0.12	0.02–0.34	0.050	2.09	4.54	1.1	3.67
June	812	18.45	0.49	6.2	0.891	0.39	21-22	885	0	-	0.045	0.07	0.00	0.1	0.18
Overall	12472	25.45	0.56	-	-	0.41	21-22	13599	0.32	0.22–0.44			8.14		

For each month (i e, sampling period): number of captured mosquitoes (*Mosq capt*); human biting rate (*HBR*); proportion of parous females (*Q*); duration of the gonotrophic cycle (*g*) in days; survival rate (*p*); proportion of endophagous females (*Endo*); median hour of biting activity (*Median hour*), i e, hour at which 50% of mosquitoes have already bitten; number of mosquitoes processed by PCR to search for *Plasmodium* parasites (*Mosq PCR*); sporozoite rate (*s*) and its 95% confidence interval; estimates of the human feeding rate (*a*); estimates of the vectorial capacities *CV*; entomological inoculation rate (*EIR*); infective life time (in days), and density of infective females.

### Estimation of the human feeding rates (*a*)

The human feeding rate was computed as the human blood index (HBI) divided by the duration (in days) of the gonotrophic cycle *g*[24]. The HBI has been estimated earlier for *An. pseudopunctipennis* and is ≈ 0.3 in Mataral (cf Table two of [44]), but may range 0.3-05 depending on the location [44]. To estimate the relative importance of HBI for *An. argyritarsis* as compared to *An. pseudopunctipennis*, a series of four experiments were carried out in May 2005, June 2005, April 2006 and June 2006. In each experiment, animal bait was disposed outdoors under mosquito net traps and mosquitoes were regularly sampled during the night. Each experiment was Latin-square designed. Four bait types were used: man (under double mosquito net to avoid mosquito bites), donkey, sheep, and goats. With the Latin-square design and these four baits, one experiment lasted four nights, during which the bait was swapped to nets every night according to the statistical design. Trophic preferences were measured by the forage ratio index [45] which simply compares the percent of use with the percent of abundance. Forage ratio index were computed for each vertebrate *i* as the percent of engorged mosquitoes which have fed upon the species *i* divided by the percent which it comprises of the total population of hosts available in the mosquito’s habitat [46]. Forage ratios, their standard errors and 95% confidence intervals were computed as in [44]. In each series of experiments and for each of the two *Anopheles* species, the hypothesis that mosquitoes were selecting resources at random was tested using a G-test [47].

### Estimation of the sporozoite rates (*s*)

The identification of *Plasmodium* in wild-caught mosquitoes was carried out using the Chelex-based DNA extraction protocol and the semi-nested multiplex PCR described in [2]. The primers used were able to detect *P. vivax, Plasmodium falciparum* and *Plasmodium malariae*, the three possible species present in Bolivia. The protocol enabled to group mosquitoes in pools of 1 to 20 individuals to limit the number of PCR runs, without loss of sensibility in parasite detection [2]. Monthly sporozoite rates were computed for each locality using the R-package binGroup [48] which uses algorithms that take into account the pooling of insects to estimate the prevalence of infection.

### Meteorological data

The following climatic parameters were taken into account to compare the dynamics of adult densities between the two species: the mean temperature one month before sampling, the amount of precipitation one month before sampling and the mean relative humidity one month before sampling. Evapo-transpiration also influences mosquito population dynamics [49] so the mean potential evapo-transpiration (PET) one month before sampling was also taken into account. PET was estimated by the Prescott formula [50] which provides reliable results in comparison to other empirical equations [51]. For the present study, the Prescott formula has been developed using [51] and [52] and the following equation was obtained:

$$\text{PET}=K.{\left[d.{\left(A-\frac{B}{T_{\text{moy}}+C}\right)}^{10}.\left(1-H_{\text{moy}}\right)\right]}^{0.75}$$

This equation depends on basic climatologic measurements (*T*_*moy*_: mean temperature, *H*_*moy*_: mean humidity during the study period), the three constants (*A*, *B* and *C*) of Antoine's formula [52], *d*: a constant which depends on the number of days of computation [51], and a coefficient *K*, characteristic of the local vegetation. For the present study, *K* was set to 1, which is the value attributed to shrubs or bare soils landscapes [51] such as those found in the two studied sites. Data on daily precipitation, temperature and relative humidity were obtained from nearby field meteorological stations of the Bolivian national meteorological service (SENAMHI).

## Results

### Geographical co-existence of the two species

**Figure 1:**
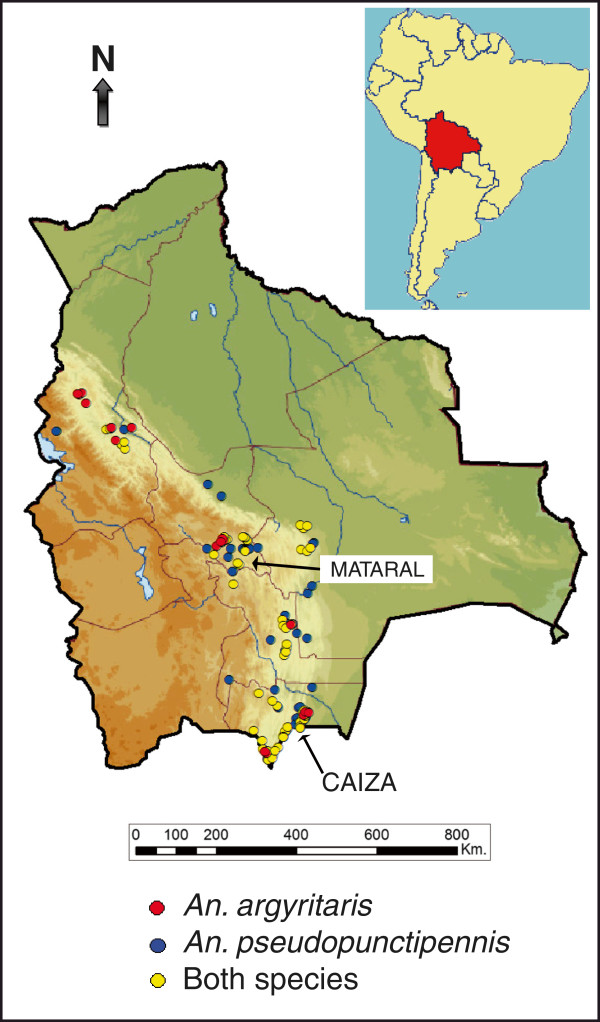
**Localization of mosquito sampling stations for *Anopheles pseudopunctipennis *and *Anopheles argyritarsis *in Bolivia.** Red dots: *An. argyritarsis* without *An. pseudopunctipennis*; blue dots: *An. pseudopunctipennis* without *An. argyritarsis*; yellow dots: both species present. Due to map scale, various larval breeding sites overlap and not all the 346 locations are visible. Mataral and Caiza where the longitudinal studies were carried out are also indicated.

From the 535 sampling sites throughout Bolivia, *An. pseudopunctipennis* and/or *An. argyritarsis* were found in 346 larval sites. They mapped the geographical distribution of both species which appeared to be characteristic of the slopes of the Andes (Figure 1). *An. pseudopunctipennis* was found without *An. argyritarsis* in 122 breeding sites, *An. argyritarsis* without *An. pseudopunctipennis* in 112 sites, and the species were sympatric in 112 sites giving a Jaccard Index of 0.323. From [28], the significant value for co-existence was 0.291 (i e, the expected random value for 350 samples at *P* = 0.05). Because the Jaccard Index was >0.291, the hypothesis that the two species share the same sites (i e, the difference is random) cannot be rejected.

*Anopheles pseudopunctipennis* was present in 67.6% of the samples while *An. argyritarsis* in 64.7%. These two proportions were not statistically different (χ^2^ = 0.65, df = 1, *P* = 0.42) indicating that both species occur with the same frequency.

In the 346 larval sites, other *Anopheles* species were associated with *An. pseudopunctipennis* and/or *An. argyritarsis* and were: *Anopheles rangeli* (39 sites), *Anopheles triannulatus s.l.* (21 sites), *Anopheles trinkae* (13 sites), *Anopheles benarrochi s.l.* (eight sites), *Anopheles nuneztovari s.l.* (seven sites), *Anopheles oswaldoi s.l.* (seven sites), *Anopheles albitarsis s.l.* (four sites), *Anopheles strodei* (three sites), *Anopheles forattinii/Anopheles costai* (two sites) and *Anopheles boliviensis* (two sites). In some of these sites, more than one associated species was present. All these associated species were captured below 880 m. Only *An. boliviensis* was found above 880 m but not above 1400 m. Above 1400 m, only *An. argyritarsis* or *An. pseudopunctipennis* were found. The minimum altitude where *An. pseudopunctipennis* was found was 206 m and for *An. argyritarsis,* 348 m*.* The maximum altitudes were 2732 m and 2323 m for *An. pseudopunctipennis* and *An. argyritarsis,* respectively.

*Anopheles pseudopunctipennis* was always found in sun-exposed, clear, slow-running water, almost always associated with filamentous algae. Typical larval breeding sites were river margins and along river banks, resurgences of water, springs and pools. *Anopheles argyritarsis* shared the same types of site but was also found in stagnant pools of various sizes (sometimes large), swamp areas (not always close to rivers and not always fully exposed to sun), as well as small temporary sites such as animal tracks and small collection of clear water in ditches, in association with grass. *Anopheles argyritarsis* has also been collected in peridomestic sites such as used tyres, flower pots, 200-litre drums, etc. (Additional file 1).

### Environment and climatic variables during the study period in Mataral and Caiza

**Figure 2:**
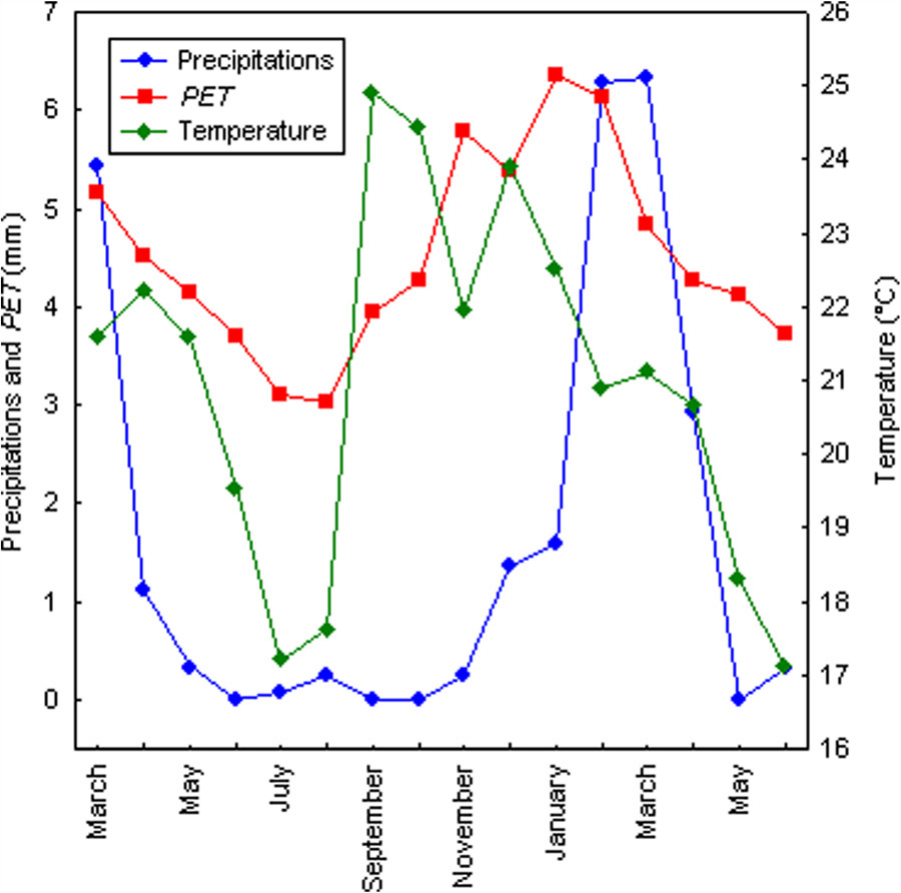
**Climatic conditions in Mataral during the study period (March 2005–June 2006).** Precipitation and potential evapo-transpiration are shown in mm. Temperatures in °C.

During the study period in Mataral, the PET was almost higher than precipitation, indicating that this locality is very dry. Only in January-February the precipitation balanced the PET. Rains were very abundant in January-March. Temperatures and PET-precipitation data divided the study period into three “seasons”: a dry and cool (<20°C) season from May to July, a dry and hot season from August to December and a “rainy” and hot season from January to March (Figure 2). In Mataral, larval breeding sites of both species were the margins of a small river where resurgences of clear, slow-running water formed small pools fully exposed to sun. These breeding sites were active mainly during the rainy season, and almost dried out during the dry season where their total surface diminished drastically. The stability of this type of breeding site was dependent on the river floods, which were frequent from November to March, according to precipitation.

**Figure 3:**
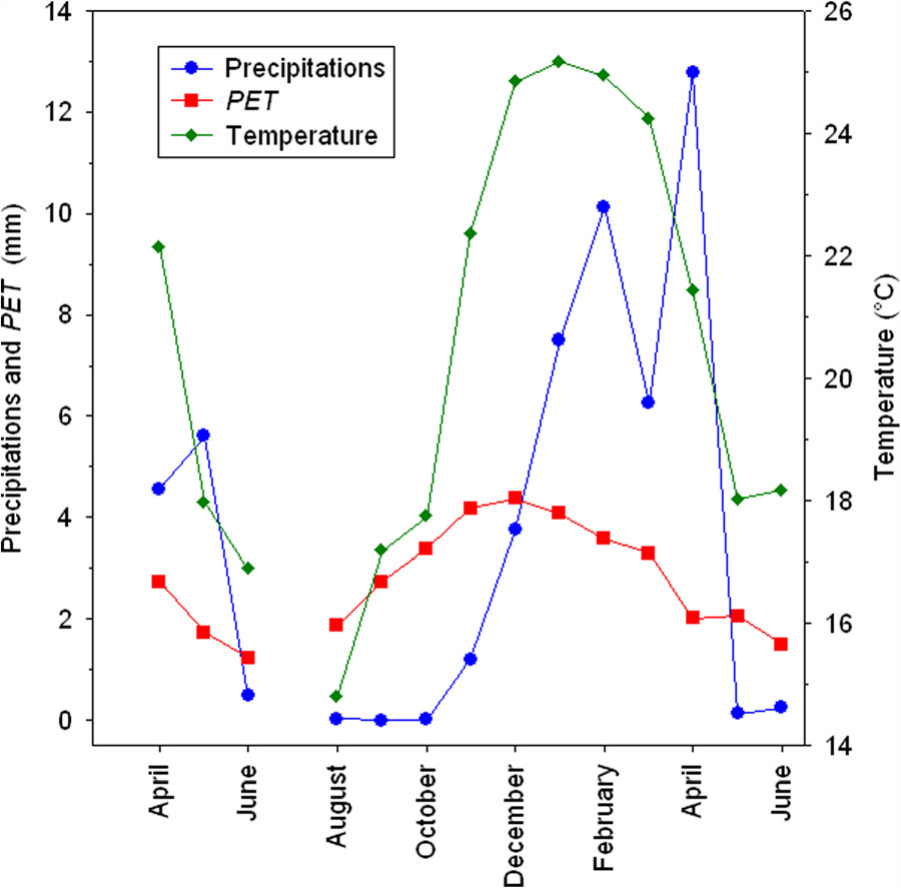
**Climatic conditions in Caiza during the study period (April 2005–June 2006).** Precipitation and potential evapo-transpiration are shown in mm. Temperatures in °C.

In Caiza, the dry period lasted from May to November and during the remaining months precipitation largely exceeded the PET. Precipitation was high from December to April. Temperatures were lower during the “dry” season. The study period can be divided into two “seasons”: a dry and cool season from May to November and a hot and humid season from December to April (Figure 3). *Anopheles pseudopunctipennis* was found only in the nearby river margins and small pools in the banks of the river while *An. argyritarsis* was found not only in association with *An. pseudopunctipennis* but also in pools of various sizes and swamp areas close to the village and independent of the river system, in animal tracks and in some peridomestic sites (used tyres, flower pots, 200-litre plastic drums, and various other small domestic containers).

### Human biting rates and relationships with climatic variables

In Mataral, a total of 12,472 *An. pseudopunctipennis* and 810 *An. argyritarsis* were captured. In Caiza, there were 2,897 and 482, respectively. In Mataral, only *An. pseudopunctipennis* and *An. argyritarsis* were present. In Caiza, other *Anopheles* species were captured: *Anopheles triannulatus s.l.*, *Anopheles benarrochi s.l.*, *Anopheles nuneztovari s.l.*, *Anopheles rangeli* and *Anopheles oswaldoi s.l.*, but always in very low numbers (monthly range: 0 – 10 individuals). HBR estimates for *An. pseudopunctipennis* and *An. argyritarsis* in Mataral are shown in Tables 1 and 2. Estimates for Caiza are shown in Tables 3 and 4.

**Table 2:** **Transmission parameters for *Anopheles argyritarsis *in Mataral during the study period (March 2005–June 2006)**

**Sampling period**	**Mosq capt**	**HBR**	**Q**	***g***	***p***	**Endo.**	**Median hour**	**Mosq PCR**	***s***	***a***	**CV**	**EIR**	***Infec life time***	***Density infective females***
March	19	1.06	-	4.2		-	-	5	0	-	-	0	-	
April	20	0.83	-	3.9		-	-	14	0	-	-	0	-	
May	246	6.15	0.47	4.6	0.849	0.33	18-19	230	0	0.024	0.90	0	0.4	0.41
June	243	7.59	0.43	5.3	0.853	0.26	18-19	218	0	0.021	0.99	0	0.2	0.29
July	29	0.69	0.48	9.2	0.923	-	-	9	0	0.012	0.10	0	0.6	0.04
August	32	1.00	0.40	6.4	0.866	-	-	13	0	0.017	0.12	0	0.2	0.03
September	1	0.17	-	3.1		-	-	-	-	-	-	-	-	
October	9	0.28	-	3.5		-	-	3	0	-	-	0	-	
November	1	0.03	-	3.9		-	-	-	-	-	-	-	-	
December	1	0.03	-	3.6		-	-	2	0	-	-	0	-	
January	4	0.13	-	4.0		-	-	4	0	-	-	0	-	
February	0	0.00	-	4.4		-	-	-	-	-	-	-	-	
March	0	0.00	-	4.5		-	-	-	-	-	-	0	-	
April	24	0.67	0.90	5.2	0.980	0.25	19-20	22	0	0.021	0.70	0	34.2	0.47
May	128	3.56	0.55	6.0	0.905	0.26	20-21	232	0	0.018	0.66	0	0.7	0.25
June	53	1.20	-	6.2	-	0.27	19-20	68	0	-	-	0	-	
Overall	810	1.65	0.48	-	-	0.30	18-19	820	0	-	-	0		

For each month (i e, sampling period): number of captured mosquitoes (*Mosq capt*); human biting rate (*HBR*); proportion of parous females (*Q*); duration of gonotrophic cycle (*g*) in days; survival rate (*p*); proportion of endophagous females (*Endo*); median hour of biting activity (*Median hour*), i e, hour at which 50% of mosquitoes have already bitten; number of mosquitoes processed by PCR to search for *Plasmodium* parasites (*Mosq PCR*); sporozoite rate (*s*); estimates of the human feeding rate (*a*); estimates of the vectorial capacities CV ; entomological inoculation rate (*EIR*); infective life time (in days).

**Table 3:** **Transmission parameters for *Anopheles pseudopunctipennis *in Caiza during the study period (April 2005–June 2006)**

**Sampling period**	**Mosq capt**	**HBR**	**Q**	***g***	***p***	**Endo.**	**Median hour**	**Mosq PCR**	***s***	***a***	**CV**	**EIR**	***Infec life time***	***Density infective females***	**Sampling period**
April	326	10.52	0.66	4.5	0.911			318	0	-	0.067	2.23	0	2.8	3.09
May	616	15.79	0.67	7.1	0.945	0.35	23-24	609	0	-	0.042	4.69	0	3.5	6.26
June	171	5.52	0.56	10.1	0.944	0.37	19-20	231	0	-	0.030	0.88	0	1.3	1.69
August	161	6.19	0.45	8.8	0.913	0.15	20-21	179	1.19	0.14–4.13	0.034	0.24	0	0.0	0.64
September	79	2.47	0.58	5.9	0.912	0.27	19-20	99	1.03	0.03–5.20	0.051	0.53	7.37	0.3	0.97
October	358	11.19	0.65	6.0	0.930	0.18	21-22	372	0.27	0.01–1.40	0.050	3.42	2.54	2.1	4.91
November	418	13.06	0.70	4.4	0.922	0.31	22-23	470	0	-	0.068	3.68	3.02	4.0	4.37
December	305	9.53	0.75	3.3	0.915	0.42	23-24	323	0	-	0.092	3.54	0	4.5	3.39
January	42	1.31	0.68	3.2	0.885	0.50	0-1	42	0	-	0.095	0.19	0	2.3	0.25
February	45	1.41	0.86	.1	0.953	0.53	23-24	45	0	-	0.095	1.29	0	12.7	0.65
March	127	3.97	0.67	3.3	0.884	0.26	0-1	126	0	-	0.092	0.44	0	2.2	0.59
April	97	3.03	0.47	4.9	0.856	0.21	22-23	95	0	-	0.062	0.07	0	0.4	0.19
May	126	3.94	0.50	7.7	0.914	0.45	20-21	125	0.83	0.03–4.22	0.039	0.16	0	1.0	0.36
June	26	1.08	0.68	7.1	0.947	0.23	20-21	29	0	-	0.042	0.09	3.27	3.1	0.12
Overall	2897	6.60	0.64			0.28	22-23	3063	0.16	0.05–0.39	0.034	0.24	1.06		

For each month (i e, sampling period): number of captured mosquitoes (*Mosq capt*); human biting rate (*HBR*); proportion of parous females (*Q*); duration of the gonotrophic cycle (*g*) in days; survival rate (*p*); proportion of endophagous females (*Endo*); median hour of biting activity (*Median hour*), i e, hour at which 50% of mosquitoes have already bitten; number of mosquitoes processed by PCR to search for *Plasmodium* parasites (*Mosq PCR*); sporozoite rate (*s*) and its 95% confidence interval; estimates of the human feeding rate (*a*); estimates of the vectorial capacities *CV*; entomological inoculation rate (*EIR*); infective life time (in days), and density of infective females.

**Table 4:** **Transmission parameters for *Anopheles argyritarsis *in Caiza during the study period (April 2005–June 2006)**

**Sampling period**	**Mosq capt**	**HBR**	**Q**	***g***	***p***	**Endo**	**Median hour**	**Mosq PCR**	***s***	***a***	**CV**	**EIR**	***Infec Life time***	***Density Infective females***
April	8	0.26	-	4.5	-	-	-	4	0	-	-	0	-	-
May	4	0.10	-	7.1	-	0.42	21-22	-	-	-	-	-	-	-
June	65	2.10	-	10.1	-	0.11	19-20	16	0	-	-	0	-	-
August	25	0.96	-	8.8	-	-	-	17	0	-	-	0	-	-
September	10	0.31	-	5.9	-	-	-	9	0	-	-	0	-	-
October	10	0.31	-	6.0	-	-	-	10	0	-	-	0	-	-
November	3	0.09	-	4.4	-	-	-	-	-	-	-	-	-	-
December	3	0.09	-	3.3	-	-	-	-	-	-	-	-	-	-
January	14	0.44	-	3.2	-	0.00	20-21	-	-	-	-	-	-	-
February	49	1.53	-	.1	-	0.13	20-21	34	0	-	-	0	-	-
March	45	1.41	-	3.3	-	0.24	20-21	11	0	-	-	0	-	-
April	100	3.13	-	4.9	-	0.24	19-20	72	0	-	-	0	-	-
May	109	3.41	0.38	7.7	0.882	0.27	19-20	100	0	0.023	0.57	0	0.3	0.12
June	37	1.54	0.61	7.1	0.933	0.14	18-19	20	0	0.014	0.69	0	1.5	0.09
Overall	482	1.10	0.53			0.20	19-20	293	0			0		

For each month (i e, sampling period): number of captured mosquitoes (*Mosq capt*); human biting rate (*HBR*); proportion of parous females (*Q*); duration of gonotrophic cycle (*g*) in days; survival rate (*p*); proportion of endophagous females (*Endo*); median hour of biting activity (*Median hour*), i e, hour at which 50% of mosquitoes have already bitten; number of mosquitoes processed by PCR to search for *Plasmodium* parasites (*Mosq PCR*); sporozoite rate (*s*); estimates of the human feeding rate (*a*); estimates of the vectorial capacities *CV*; entomological inoculation rate (*EIR*); infective life time (in days).

In Mataral, the overall mean HBR of *An. pseudopunctipennis* and *An. argyritarsis* was 25.45 and 1.65, respectively, indicating that a human may receive ≈ 15 more bites from *An. pseudopunctipennis* than from *An. argyritarsis*. In fact, the monthly HBRs of *An. pseudopunctipennis* were always greater that those of *An. argyritarsis*, with ratios ranging from four to 423 times in favour to *An. pseudopunctipennis*. Only on four occasions was the HBR of *An. pseudopunctipennis* <3 (November, December 2005, February and March 2006). In most occasions, its HBR was >10-20 indicating that this species may bite humans in high densities. On the contrary, the overall low value for *An. argyritarsis* indicated that this species, if vector, may not be able to transmit all year long, but only during some short periods. The monthly HBRs of *An. argyritarsis* were close to 0. Only during the months of May and June, was its HBR >1, reaching values >6.

In Caiza the overall mean HBR for *An. pseudopunctipennis* and *An. argyritarsis* was 6.60 and 1.10, respectively (ratio ≈ 6). The minimum and maximum ratios were 0.7 and 154, respectively, almost always in favour of *An. pseudopunctipennis*. On only two occasions *An. argyritarsis* bit humans more than *An. pseudopunctipennis*: in February and June 2006, but the HBRs were low, only 1.53 for *An. argyritarsis* (*vs* 1.41 for *An. pseudopunctipennis*) in February and 1.54 (*vs* 1.08), respectively in June, indicating a real scarcity of both species during these periods, and likely no significant difference between these low HBRs. All year long *An. argyritarsis* exhibited low values of HBR, always <3 and almost <1.5. Between July 2005 and January 2006, the HBR for *An. argyritarsis* was <1. *Anopheles pseudopunctipennis* exhibited high values of HBR, ranging from >1 to >15 with more than half of the observations >5.

**Figure 4:**
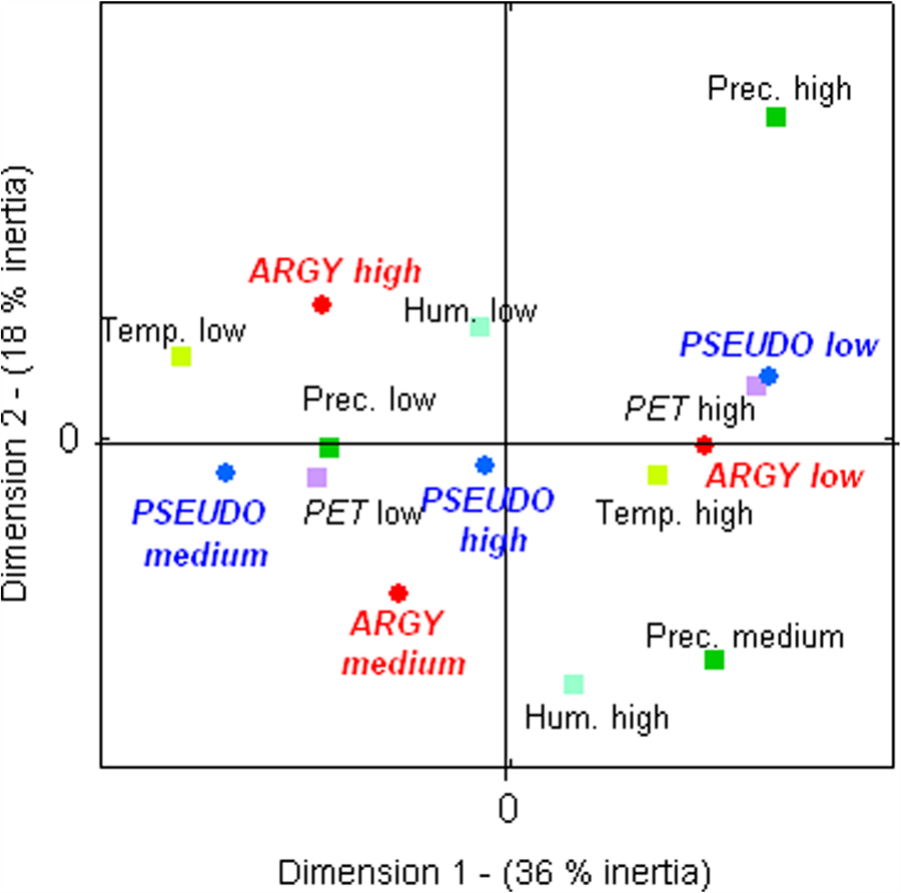
**Multiple correspondence analysis of climatic variables and human biting rate in Mataral.** Plan 1 × 2. Variables are: biting rates for *An. pseudopunctipennis* (PSEUDO) and *An. argyritarsis* (ARGY), temperature (temp), humidity (hum), potential evapo-transpiration (PET) and precipitation (precip). Variables where divided into disjointed classes of increasing values as: low, medium and high.

The MCA analysis showed that in Mataral, the population dynamics of *An. pseudopunctipennis* and *An. argyritarsis* respond in an almost identical manner to the environmental variables: both species showed high or low levels of abundance with the same ecological conditions (Figure 4). The absence of mosquitoes seemed to be particularly dependant of the high level of precipitation. High levels of mosquito densities were observed when the climatic conditions permitted a relative stability of the breeding sites (i e, when the level of precipitation was low or medium). *Anopheles argyritarsis* densities were dependant on humidity/precipitation conditions, with higher values at the end of the rainy season in May to June (Table 2). Densities of *An. argyritarsis* were low during the “dry and hot season”. *Anopheles pseudopunctipennis* was abundant all year long, but with dramatic fluctuations from one month to another.

**Figure 5:**
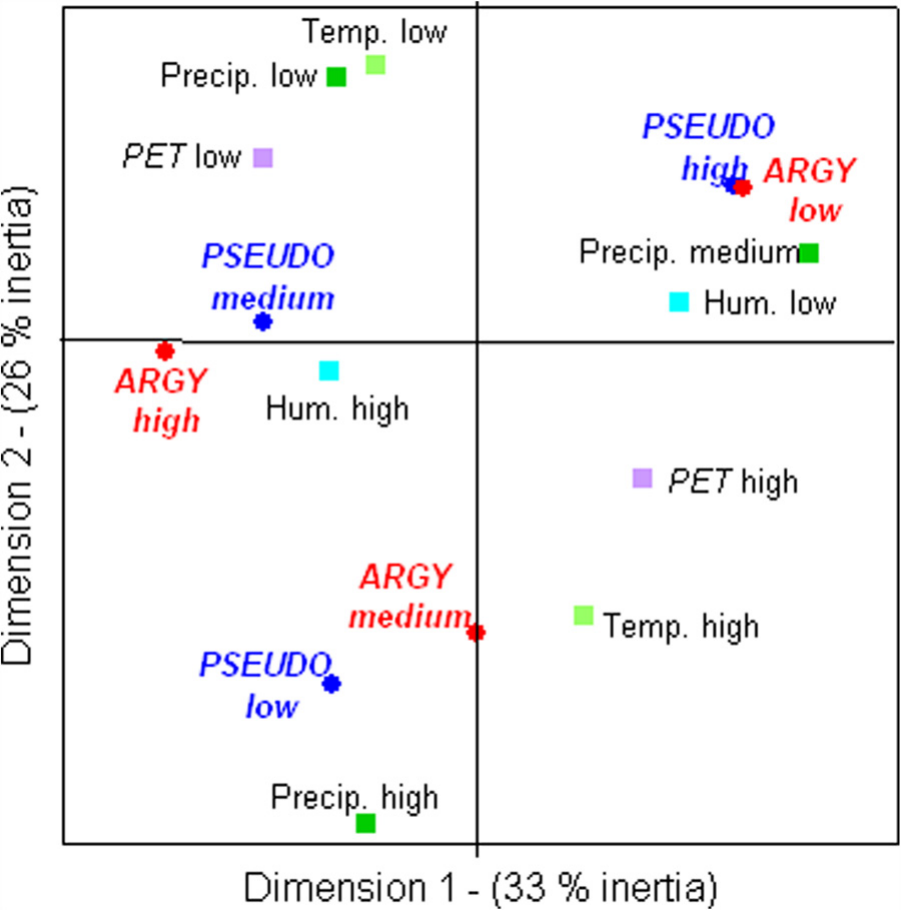
**Multiple correspondence analysis of climatic variables and human biting rate in Caiza.** Plan 1 × 2. Variables are: biting rates for *An. pseudopunctipennis* (PSEUDO) and *An. argyritarsis* (ARGY), temperature (temp), humidity (hum), potential evapo-transpiration (PET) and precipitation (precip). Variables where divided into disjointed classes of increasing values as: low, medium and high.

In Caiza, a different situation occurred. The MCA results indicated that the population dynamics of *An. argyritarsis* were the opposite of *An. pseudopunctipennis* (Figure 5)*. An. pseudopunctipennis* seems to disappear when precipitation is high. Regression analysis showed a negative correlation between the HBR of *An. pseudopunctipennis* and the level of precipitation (β = −0.74; *P* = 0.036). *Anopheles argyritarsis* was more abundant during “humid” periods (i e, high level of relative humidity and low value for PET). Regression analysis showed a positive correlation between the abundance of *An. argyritarsis* and the level of precipitation (β = 0.852, *P* = 0.007). The relative humidity (β = 0.739, *P* = 0.002) was positively linearly linked to *An. argyritarsis’* HBR while PET (β = −0.59, *P* = 0.026) was negatively correlated, indicating their influence on mosquito survival.

### Endo-exophagy

*Anopheles pseudopunctipennis*, although not very endophagous, exhibited a more endophagous pattern activity than *An. argyritarsis* (Tables 1, 2, 3 and 4). In Mataral, the overall proportion of endophagous *An. pseudopunctipennis* was 0.41 (12,581 mosquitoes captured; 5,213 endophagous), and only 0.30 for *An. argyritarsis* (828 mosquitoes captured; 246 endophagous). These two proportions were statistically different (χ^2^ = 43.7; df = 1; *P* = 0). At each sampling occasion, the proportion of endophagous *An. pseudopunctipennis* was always higher than that of *An. argyritarsis,* although below 0.5. An identical situation occurred in Caiza where the overall proportion of endophagous *An. pseudopunctipennis* was 0.28 (3,042 mosquitoes captured; 843 endophagous), and 0.20 for *An. argyritarsis* (516 mosquitoes captured; 102 endophagous). The two proportions were again statistically different (χ^2^ = 13.9; df = 1; *P* = 0). In both localities, the ratio between the two species favours *An. pseudopunctipennis,* which appeared to be 1.4 more endophagous than *An. argyritarsis*.

### Hourly biting patterns

The median time of capture (i e, the moment in the night when 50% of the total number mosquitoes were captured) is given in Tables 1, 2, 3 and 4. Both *Anopheles* species appeared to be early-biting species: the median times for *An. pseudopunctipennis* were 21.00-22.00 and 22.00-23.00 in Mataral and Caiza, respectively. *Anopheles argyritarsis* bites earlier, almost at dusk. Its median times of capture were 18.00-19.00 and 19.00-20.00 in Mataral and Caiza, respectively.

### Survival rates (*p*)

In both localities, captures of *An. pseudopunctipennis* were always sufficient to compute survival rates using the Davidson’s equation. However, for *An. argyritarsis*, densities were too low during several months to give accurate estimates. Moreover, dissections of mosquitoes have not been carried out for each sampling period. Comparison of survival rates amongst the two species was only feasible in May 2005, June 2005 and May 2006 in Mataral and May 2006 in Caiza. Results are given in Tables 1, 2, 3 and 4.

Parous females are epidemiologically dangerous as they may transmit parasites if they have lived long enough for the parasite to complete its extrinsic cycle. For *An. pseudopunctipennis*, the proportion of parous females ranged 50–60%, and only 35–55% for *An. argyritarsis.* On all occasions, the proportion of parous females was lower for *An. argyritarsis* than for *An. pseudopunctipennis*. However, these proportions were not statistically different amongst the two species (except in June 2005). Then, if it is assumed that the gonotrophic cycle duration of both species is likely to be the same, the survival rates of both species are likely to be identical. When a difference is observed in the parous rate, as in June 2005, it is in favour of *An. pseudopunctipennis* and therefore, the general tendency is that the survival rate of *An. argyritarsis* may be slightly lower than that of *An. pseudopunctipennis*.

### Human feeding rates (*a*)

Forage ratios, standard errors and 95% confidence intervals are given in Table 5. For *An. pseudopunctipennis*, forage ratios for “man” were always >1 indicating that this *Anopheles* species is attracted to man. Other vertebrates, such as sheep and donkey, were also attractive. However, the 95% confidence intervals indicated that this preference is not always marked. On only two occasions the *G*-tests indicated that *An. pseudopunctipennis* chose amongst the baits (man and donkey in May 2005, and man and sheep in June 2005). *Anopheles argyritarsis* was never attracted to man (forage ratios always <1, and 95% confidence intervals almost below the value 1). This *Anopheles* species was instead attracted to animals (sheep and donkey in particular) indicating a more pronounced zoophilic attitude than *An. pseudopunctipennis.* In the four experiments, the ratio between the proportion of human-fed mosquitoes in *An. pseudopunctipennis* and *An. argyritarsis* ranged between 2–5 with an overall computed value of 2.7.

**Table 5:** **Forage ratios for *Anopheles pseudopunctipennis *and *Anopheles argyritarsis***

**Experiment**	**Species**	**% that fed on man**	**Forage ratios**				
			**Man**	**Sheep**	**Donkey**	**Goat**	**G-test**
May 2010	*An. pseudopunctipennis*	38.1%	1.53 (0.17)	0.55 (0.12)	[0.31– 0.78]	0.58 (0.12)	26.6 (*P* < 0.05)
			[1.19-1.86]	[0.31– 0.78]	[1.02–1.67]	[0.34–0.82]	
	*An. argyritarsis*	7.1%	0.28 (0.27)	0 (−)	3.42 (0.37)	[2.69–4.16]	-
			[0–0.82]	[−]	[2.69–4.16]	[0–0.82]	
June 2010	*An. pseudopunctipennis*	38.5%	1.15 (0.10)	1.46 (0.11)	-	0.39 (0.07)	47.9 (*P* < 0.05)
			[0.96-1.35]	[1.25–1.66]		[0.25–0.52]	
	*An. argyritarsis*	20.6%	0.62 (0.21)	1.42 (0.26)	-	0.97 (0.24)	3.6 (*P* = 0.16)
			[0.33–1.42]	[1.43–2.56]		[0–0.36]	
April 2011	*An. pseudopunctipennis*	44.0%	1.32 (0.21)	1.02 (0.20)	0.66 (0.17)	-	3.75 (*P* = 0.15)
			[0.91–1.73]	[0.62–1.41]	[0.31–1.00]		
	*An. argyritarsis*	10.5%	0.32 (0.21)	1.42 (0.34)	1.26 (0.34)	-	5.4 (*P* = 0.06)
			[0–0.73]	[0.75–2.09]	[0.60–1.92]		
June 2011	*An. pseudopunctipennis*	25.0%	1.00 (0.33)	0.43 (0.23)	2.43 (0.37)	0.14 (0.14)	21.2 (*P* < 0.05)
			[0.36–1.64]	[0–0.88]	[1.70–3.15]	[0–0.42]	
	*An. argyritarsis*	0.0%	0 (−)	1.00 (0.86)	3.00 (0.86)	0 (−)	-
			[−]	[0–2.70]	[1.30–4.70]	[−]	
Overall	*An. pseudopunctipennis*	38.1%					
		*n* = 417					
	*An. argyritarsis*	14.1%					
		*n* = 71					

Proportion of mosquitoes that fed on man, forage ratios (error standard in parenthesis; 95% confidence intervals in brackets) for each of the vertebrate baits during the four mosquito-net experiments, and the associated G-tests with their probability *P*. A forage ratio >1 indicates preference, while <1 indicates avoidance. For the G-tests, when *P* < 0.05 the null hypothesis of equal use of resources can be rejected. *n* = total number of mosquitoes captured during the four experiments (overall).

The overall computation showed that 38% of *An. pseudopunctipennis* fed on man (and therefore 62% fed on animals) while only 14% of *An. argyritarsis* fed on man (and therefore 86% fed on animals). These two proportions where statistically significant (χ^2^_(1)_ = 8.65; *P* = 0.003). *Anopheles pseudopunctipennis* took 2.7 times more blood meals on man than *An. argyritarsis*. If, in a first approach, it can be considered that the duration of the gonotrophic cycle is identical for the two *Anopheles* species, the parameter *a* of the vectorial capacity is 2.7 (i e, ≈3) times higher for *An. pseudopunctipennis* than for *An. argyritarsis*. If the duration of the gonotrophic cycle is not identical in the two *Anopheles* species, the cycle for *An. argyritarsis* would have to be ≈ 3 times shorter than that of *An. pseudopunctipennis* to give an identical value for *a*, which is biologically impossible. Estimates of *a* are given in Tables 1, 2, 3 and 4 for *An. pseudopunctipennis* and *An. argyritarsis* in Mataral and Caiza, respectively. An estimated HBI of 0.3 was used for *An. pseudopunctipennis*, and an estimated HBI value of 0.3/2.7 = 0.11 was assumed for *An. argyritarsis*.

### Duration of the extrinsic period of *Plasmodium vivax (n)*

Taking into account the mean ambient temperature observed during the sampling periods in both localities, the duration of the extrinsic period of *P. vivax* ranged 10.1–40.2 days in Mataral and 10.3–99.4 days in Caiza (Table 6). The coldest months exhibited high values almost incompatible with mosquito survival.

**Table 6:** **Mean monthly temperature (*Tm*) and duration of the extrinsic cycle (*n*)**

**Month**	**Mataral**		**Caiza**	
	***Tm***	***n***	***Tm***	***n***
March	21.6	14.8		
April	22.5	13.2	21.7	14.6
May	20.9	16.5	18.1	29.3
June	19.6	20.5	16.8	45.5
July	15.9	37.0		
August	18.7	24.8	15.6	99.4
September	24.9	10.1	17.1	40.6
October	23.7	11.4	18.5	26.1
November	22.3	13.5	22.0	14.0
December	23.5	11.7	24.6	10.4
January	22.2	13.6	24.7	10.3
February	21.0	16.2	24.7	10.3
March	21.3	15.5	24.4	10.6
April	20.4	17.9	20.5	17.5
May	18.5	26.6	18.4	26.9
June	17.1	40.2	17.7	32.5

Values are computed for Mataral and Caiza for the study period (March 2005–June 2006). The mean monthly temperatures in °C and durations of the extrinsic cycle of *Plasmodium vivax* in days.

### Sporozoite rates *(s)*

Results for both species and both localities are given in Tables 1, 2, 3 and 4. Only *P. vivax* was detected in *An. pseudopunctipennis* in Mataral and Caiza. In both localities, prevalence of infection was low, almost <1%. No *Plasmodium sp*. was detected in any of the PCR-processed *An. argyritarsis* from both localities, and none of the other *Anopheles* captured in Caiza were found positive.

Infection prevalence of *An. pseudopunctipennis* from Mataral ranged 0–1.63% (Table 1). Parasites were detected almost all year long in low prevalence (*s* < 0.5%). The overall prevalence of infection computed on 1,374 pools of mosquitoes totalling 13,599 individuals, was 0.32%. In Caiza (Table 3), the prevalence of infection of *An. pseudopunctipennis* ranged 0–1.19%. Again, monthly prevalence was low, even 0 for most of the months sampled. In only four months was the prevalence of infection positive. However, because low numbers of mosquitoes were captured in Caiza, the chance of detecting positive was low. Transmission was more intense (*s* >0) during the months of September, October and November 2005. In May 2006, few mosquitoes were detected positive (*s* = 0.83%). The overall prevalence estimated from 421 pools of mosquitoes totalling 3,063 individuals in Caiza, was 0.16%, no difference to the value computed in Mataral.

### Estimation of vectorial capacities, EIRs and derived statistics

Values are shown in Tables 1, 2, 3 and 4. In Mataral, for *An. pseudopunctipennis* (Table 1), the expected infective life time ranged 0.1–29.7 days and was <1 day during the cool and dry season. During the warmer season the value was about two days, and even around five days, giving time for the mosquito to transmit. The density of infective *An. pseudopunctipennis* females ranged 0.18 to 23.32 with no marked seasonal fluctuations. The EIR was almost >0, and when positive, ranged 2.4 to 28.4 indicating that this mosquito, in this locality, was a relatively efficient malaria vector, in particular after the rainy season (i e, from March to June). In general terms, the vectorial capacity fluctuated and no particular season was detected for potential transmission (i e, vectorial capacity >1). On the contrary, in Mataral for *An. argyritarsis* the EIR was 0 all year long (Table 2). The expected infective life time was always <1 day, except in April 2005. The density of infective females was always <0.5.

In Caiza, for *An. pseudopunctipennis* (Table 3), the EIR was >0 in September–November (i.e., before the rainy season) and in June (end of the rainy season). The vectorial capacity was >1 in October to December (first year of the study) indicating the likely period of transmission. The expected infective life was almost >2 days. The density of infective females followed the CV dynamics, with high values before and at the end of the rainy season. For *An. argyritarsis*, data were sufficient to compute statistics only in May and June of the second year. However, values for the CV, the expected infective life time and the density of infective females were low, all <1 (Table 4).

## Discussion

The locations where *An. pseudopunctipennis* and *An. argyritarsis* were captured indicate that both species are characteristic of the foothills and dry valleys of the Andes of Bolivia. They are species of “altitude” as compared to the other *Anopheles* species, although they are also numerous in the lowlands close to the mountainous regions. In the two studied localities, *An. pseudopunctipennis* was always captured in higher quantities than *An. argyritarsis*, a situation similar to the one described in northern Argentina [22]. The two species may share the same larval breeding sites, such as the margin of rivers and resurgences where clear water runs slowly with filamentous algae. When this appends in a locality, the population dynamics of the two species are almost identical and depend essentially on precipitation that provokes rapid river floods that may destroy the larval breeding sites, as it was the case in the Mataral locality. In general terms, this is a general situation that occurs on the slopes of the Andes. *Anopheles argyritarsis* may colonize sites that are not river-dependent, such as swamps or artificial containers. Then precipitation may increase the number or the surface of larval breeding sites and therefore increase adult densities if survival conditions are adequate (in terms of temperature, humidity and PET), as seen in Caiza.

In the study, PET appeared as an interesting parameter that may influence mosquito population dynamics, in particular that of *An. argyritarsis,* as was also demonstrated with some African *Anopheles* species [53]. The present study was carried out in only two Bolivian localities and it would be inadequate to generalize the results to the whole country. However, *Plasmodium* parasites were not detected in *An. argyritarsis,* contrary to *An. pseudopunctipennis* below the same ecological conditions. Moreover, *An. pseudopunctipennis* always exceeded *An. argyritarsis* for densities, daily survival rate, antropophilly, expected infective life etc. making this mosquito a more efficient malaria transmitter as demonstrated by its higher values of vectorial capacity. The biting behaviour of *An. pseudopunctipennis* also favoured transmission as compared to that of *An. argyritarsis*: (i) it was more endophagic and therefore was more in contact with humans at night, and (ii) the very early biting activity of *An. argyritarsis* did not favoured transmission as it could be more easily killed when biting because humans were not sleeping when the mosquito was active.

## Conclusions

The present study could not incriminate *An. argyritarsis* as a malaria vector in the two studied localities, unlike *An. pseudopunctipennis*. Although the study could not be generalized to other Bolivian regions, it argues in favour of the non-vector status of *An. argyritarsis* and therefore agree with previous conclusions [5]. Above 1000 m of altitude, *An. pseudopunctipennis* might be the only malaria vector and priority control efforts should then be directed toward this species. Below this altitude, other *Anopheles* species might vector *Plasmodium* parasites along with *An. pseudopunctipennis*. The sampling survey carried out in Bolivia, pointed out some potential secondary vectors: *An. rangeli*, *An. triannulatus s.l. An. trinkae*, *An. nuneztovari s.l.*, *An. benarrochi s.l.* and *An. oswaldoi s.l.* are amongst the best candidates. Malaria is still a public health problem in Bolivia and some regions of the Andes, although less affected than the Amazonian region, are still the focus of the disease. Data from the present study could be used as a basis to derive transmission statistics and identify the abiotic parameters to be monitored in order to precise seasons of optimal intervention, at least directed towards *An. pseudopunctipennis*.

## Abbreviations

HBR: Human biting rate; EIR: Entomological inoculation rate; HBI: Human biting index; INLASA: Instituto Nacional de Laboratorios de Salud (La Paz, Bolivia); PCR: Polymerase chain reaction; PET: Potential evapo-transpiration; CV: Vectorial capacity; MCA: Multiple correspondence analysis.

## Competing interests

The authors declare that they have no competing interests.

## Authors’ contributions

FL designed the project, sampled and processed (dissections) the mosquitoes in the field, performed the statistical analysis and drafted the manuscript. CA and RT processed the mosquitoes in the field (dissections) and identified the malaria parasites in the laboratory (PCR). LT processed the co-occurrence data. All authors read and approved the final manuscript.

## Supplementary Material

Additional file 1 List of sampling stations where An. pseudopunctipennis and/or An. argyritarsis were collected in Bolivia. Click here for file
